# A One Health Research Framework for Animal-Assisted Interventions

**DOI:** 10.3390/ijerph16040640

**Published:** 2019-02-21

**Authors:** Karin Hediger, Andrea Meisser, Jakob Zinsstag

**Affiliations:** 1 Department of Epidemiology and Public Health, Swiss Tropical and Public Health Institute, 4002 Basel, Switzerland; andrea.meisser@meisan.ch (A.M.); jakob.zinsstag@swisstph.ch (J.Z.); 2 Department of Psychology, University of Basel, 4055 Basel, Switzerland; 3 Institute for interdisciplinary research on human-animal relationship (IEMT) Switzerland c/o Swiss Tropical and Public Health Institute, 4002 Basel, Switzerland; 4 University of Basel, 4001 Basel, Switzerland

**Keywords:** animal-assisted interventions, animal-assisted therapy, animal welfare, human-animal relationship, One Health

## Abstract

Background: The integration of animals into healthcare, referred to as animal-assisted intervention, is a rapidly growing research field and was previously related to One Health. However, the assessment of synergistic effects of animal-assisted interventions (AAI) has been poorly addressed to date. Method: We discuss experiences in integrated human and animal assessments in AAI and provide a methodical framework for One Health approaches in AAI research. We propose theoretical consideration of an integrated human and animal health assessment, as well as the use of such an integrated approach in research. Based on the existing research, we argue that, for a deeper understanding of AAI mechanisms, parallel research designs are needed. Results and Conclusion: Our paper shows that a One Health study design is necessary to ensure that a tradeoff in health of animals is prevented and that an added value, or synergistic benefit, can be achieved on both sides during animal-assisted interventions.

## 1. One Health

One Health recognizes the inextricable linkage of humans, animals and their environment [1] and is defined as any added value in terms of human and animal health and wellbeing, reduced cost, or sustained environmental services that can be achieved by a closer cooperation of human and animal health and other disciplines which could not be achieved if the sectors work separately [2]. Previous research, for example, from vaccination campaigns, shows that a One Health approach provides a clear benefit for the health of humans and animals alike [3,4,5].

We address the benefits and possibilities of a closer cooperation of human and animal health in the context of animal-assisted interventions (AAI). Earlier work relates AAI to One Health but does not address the assessment of synergistic benefits of AAI [6,7,8,9]. In this paper, we discuss experiences in integrated human and animal assessments in AAI and provide a methodical framework for One Health approaches in AAI research.

## 2. Animal-Assisted Interventions

Animal-assisted interventions subsume different interventions that incorporate human-animal teams in formal human services, referred to as animal-assisted therapy, animal-assisted education, animal-assisted coaching and animal-assisted activities [10].

Numerous studies document the significant influence of the human-animal relationship on human wellbeing and health. Although the results are sometimes contradictory [11], there is increasing evidence that interaction with animals can improve human health [12] and that AAI is an effective treatment for mental, behavioral and neurological disorders across different demographic populations [13,14,15,16,17,18,19,20]. AAI leads to enhanced physical, social and emotional wellbeing, possibly modulated via common brain networks involved in reward, emotion, affiliation [21] and the interconnection of the oxytocin system of both humans and animals [22]. Therefore, AAI is increasingly used as an adjunct in healthcare within a broad range of physical and mental health problems in hospitals, rehabilitation clinics, psychiatric facilities, prisons, schools, nursing homes and many more. 

## 3. A One Health Framework for AAI 

From a One Health perspective, ethically justifiable AAI should generate an added value in health and wellbeing for humans as well as animals and avoid any suffering in both. In the last few years, One Health has been understood to be an important framework for AAI [6,7,9,23,24], and there is growing awareness within AAI practice that the animal’s health and welfare should as well be a focus.

Internationally approved guidelines for AAI [10] are in place, and institutions in the field of human-animal interaction have defined standards and basic requirements (e.g., standards of the International Society for Animal-Assisted Therapy (ISAAT) and guidelines of the Veterinary Association for Animal Protection in Germany (TVT) [25,26]). In Italy, the legislative regulation process has a clear connection to the One Health concept [8], while in other countries, such as Sweden and Austria, legal regulations are developing. The international guidelines from the International Association of Human-Animal Interaction Organizations (IAHAIO) stipulate that all animals involved must enjoy this type of activity and not be overworked, overwhelmed or jeopardized in their safety and comfort [10]. These guidelines are squarely in line with the principles of One Health, but the question arises of how to assess joy or exhaustion in the animal during AAI. This requires evidence-based knowledge.

Most research on AAI focuses on the human side, but there is specific literature on dogs [27], horses [28] and guinea pigs [29]. To ensure that the interdependencies between human and animal health are taken into account, we propose a One Health framework for AAI research which would demonstrate under which circumstances there is no tradeoff of human benefits against animal health and wellbeing and under which circumstances animals could actually benefit from such interactions with humans. This is an ethical standard to which those who utilize animals are bound [30,31]. In addition, ensuring and fostering the health and wellbeing of the animals leads to positive rebound effects.

## 4. One Health Research Designs

We propose two broad areas of questions within this One Health framework of AAI research. On one hand, benefits and risks of AAI in the participation for humans and animals need to be identified. Applying this on the animal’s behalf implies that research identifies conditions that enable as much enrichment behavior and welfare as possible and minimize distress and health risks. This must be evaluated across different species but also take the personality of the individual animal into account [29,32]. These questions can be addressed in a sequential study design, where separate studies look at the effects on either the animals or the humans. 

Additionally, the interrelation and the reciprocal influence of the relationship between participating humans and animals during animal-assisted interventions need to be further investigated. What happens between a human and an animal? What is their relationship, and how do the individuals react to and influence each other? For these questions, parallel study designs are needed in which humans and animals are investigated simultaneously.

Most existing research addressing effects of AAI in animals uses sequential designs, investigating the animals before, during and/or after AAI, and thus provides insight on factors influencing stress and wellbeing of participating animals [32,33,34,35,36,37]. For example, client age [38], as well as the amount of work experience a dog has [39] and whether it is working off- vs. on-lead [40] significantly influences the amount of stress for dogs. A recent study investigating the effects of AAI on guinea pigs under different conditions demonstrated that the possibility for retreat and free interactions during AAI reduces stress and fosters enrichment behaviors in guinea pigs [29].

An example of parallel research design is examining salivary cortisol levels of human-dog dyads showing that both dog handlers and dogs had higher cortisol concentrations on therapy days than on control days, indicating a higher stress level [41]. Schöberl and colleagues [42] investigated the cortisol response of human-dog dyads in response to different challenging situations, concluding that both owner and dog social characteristics influenced dyadic cortisol variability. Recent research measured heart rate and heartrate variability simultaneously in horses and humans [43] and applied this approach also to assess effects during AAI [44,45,46]. However, such designs are still scarce, and more are needed to understand the reciprocal influence of the relationship between the humans and the animals taking part in interventions. 

## 5. Conclusions

Implementing a One Health framework in AAI research shows that more studies addressing the health and wellbeing of participating animals are needed. We suggest that parallel research designs are needed to improve understanding of AAI mechanisms. Moreover, future research addressing animals should focus on stress reduction as well as enhancement of positive welfare indicators to identify conditions that might provide benefits to animals participating in AAI [27]. There is a growing body of literature describing assessment of animal welfare which can be used in AAI research [27,28,47,48,49]. Future research should combine different methods to investigate the relationship between behavioral and physical outcomes and use multidimensional, systemic approaches that include environmental factors and the social context [42]. Also, studies investigating long-term consequences of AAI on the quality of life in participating animals are warranted.

The proposed One Health framework for AAI requires a transdisciplinary approach and mutual interest from both scientists and practitioners. Accordingly, a One Health framework, as proposed, could overcome differences in terminology and different discipline-specific outcomes and lead to an added-value just through capturing a One Health perspective in AAI research. An integrated One Health study design is necessary to avoid a tradeoff between human and animal health and ensure that an added value in terms of synergistic benefits can be achieved on both sides during animal-assisted interventions. More specific guidelines for AAI and human animal interaction should be developed on the basis of such evidence-based knowledge.
